# Glyphosate does not substitute for glycine in proteins of actively dividing mammalian cells

**DOI:** 10.1186/s13104-019-4534-3

**Published:** 2019-08-08

**Authors:** Michael N. Antoniou, Armel Nicolas, Robin Mesnage, Martina Biserni, Francesco V. Rao, Cristina Vazquez Martin

**Affiliations:** 1 Department of Medical and Molecular Genetics, Faculty of Life Sciences & Medicine, Gene Expression and Therapy Group, King’s College London, Guy’s Hospital, 8th Floor, Tower Wing, Great Maze Pond, London, SE1 9RT UK; 2 DC Biosciences, James Lindsay Place, Dundee, DD1 5JJ UK; 3 Present Address: IST Austria Proteomics Service, Lab Building East, Am Campus 1, 3400 Klosterneuburg, Austria; 4 Present Address: Platinum Informatics Ltd., Unit 8, The Vision Building, 20 Greenmarket, Dundee, DD1 4QB UK

**Keywords:** Glyphosate, Glycine, Proteome

## Abstract

**Objectives:**

Glyphosate (*N*-phosphonomethyl glycine) and its commercial herbicide formulations have been shown to exert toxicity via various mechanisms. It has been asserted that glyphosate substitutes for glycine in polypeptide chains leading to protein misfolding and toxicity. However, as no direct evidence exists for glycine to glyphosate substitution in proteins, including in mammalian organisms, we tested this claim by conducting a proteomics analysis of MDA-MB-231 human breast cancer cells grown in the presence of 100 mg/L glyphosate for 6 days. Protein extracts from three treated and three untreated cell cultures were analysed as one TMT-6plex labelled sample, to highlight a specific pattern (+/+/+/−/−/−) of reporter intensities for peptides bearing true glyphosate treatment induced-post translational modifications as well as allowing an investigation of the total proteome.

**Results:**

Comparative statistical analysis of global proteome changes between glyphosate treated and non-treated samples did not show significant differences. Crucially, filtering of data to focus analysis on peptides potentially bearing glycine for glyphosate replacement revealed that the TMT reporter intensity pattern of all candidates showed conclusively that they are all false discoveries, with none displaying the expected TMT pattern for such a substitution. Thus, the assertion that glyphosate substitutes for glycine in protein polypeptide chains is incorrect.

**Electronic supplementary material:**

The online version of this article (10.1186/s13104-019-4534-3) contains supplementary material, which is available to authorized users.

## Introduction

**Fig. 1:**
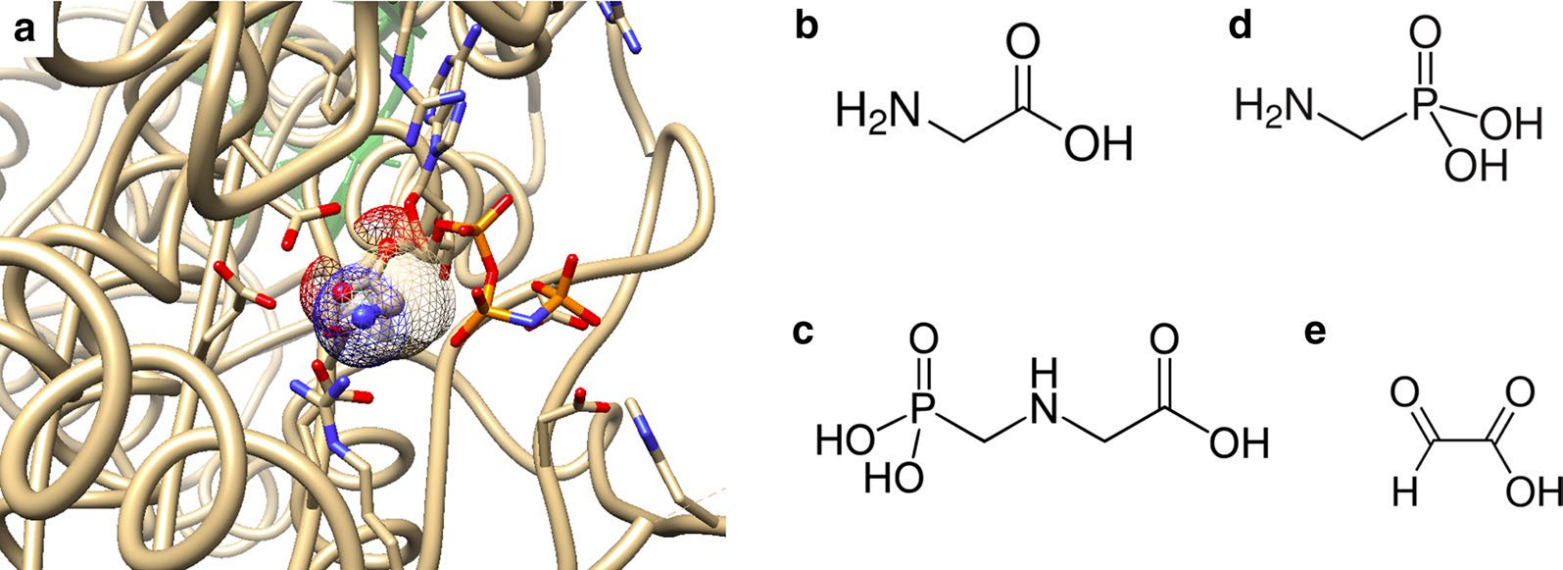
**a** Diagram of human glycyl-tRNA synthetase (brown) with tRNA (green), phosphoaminophosphonic acid-adenylate ester and glycine (ball and sphere with surface) bound at the active site (PDB4KR3). By comparison to glycine (**b**), glyphosate (**c**) is unlikely to bind to the active site due to the steric hindrance of its phosphonate group. The structure of the metabolites of glyphosate aminomethylphosphonic acid (**d**) and glyoxylate (**e**) is also presented

Glyphosate (*N*-phosphonomethyl glycine; Fig. 1) is the active ingredient in the most widely used herbicides, the best known being Roundup [1]. The safety of glyphosate and its commercial formulations has been an area of intense research. Findings include an alteration of mitochondrial function, which generates reactive oxygen species [2–4]. Oxidative stress caused by glyphosate (or Roundup) has also been found to be concomitant with DNA damage [5–8], and that antioxidants can mitigate these effects [9].

It has also been asserted that glyphosate causes toxic effects by substituting for glycine in polypeptide chains leading to protein misfolding with subsequent altered cellular biochemistry and toxic outcomes [10]. The evidence used to support this claim is twofold. First, that glyphosate can potentially form artificial N-substituted glycine polymers (“peptoids”) [11]. The synthesis of glyphosate peptoids has not been reported, but if they could be synthesised this does not provide evidence that glyphosate can be incorporated into natural polypeptides. Second, the proposers refer to unpublished studies conducted by the US-based company DuPont in which radioactively labelled ^14^C-glyphosate was administered to goats. The proposers refer to two outcomes from this goat feeding study to argue for glyphosate-glycine substitution in proteins. First, only some of the ^14^C-glyphosate was extractable from tissues of these animals. Second, digestion of liver, kidney and omental fat tissues with a mixture of proteases was able to release more ^14^C-glyphosate whilst protease treatment of muscle did not enhance release of ^14^C-glyphosate. These arguments not only ignore the apparent contradiction that protease treatment of goat tissues either does or does not lead to enhanced ^14^C-glyphosate release, but other simpler explanations such as glyphosate being adsorbed onto or trapped within proteinaceous structures. Furthermore, molecular modelling suggests that glyphosate is unlikely to bind to the active site of glycyl-tRNA synthetase due to steric hindrance by its phosphonate group (Fig. 1) and thus unable to be inserted in place of glycine during polypeptide chain elongation. We have previously addressed the validity of the claim that glyphosate substitutes for glycine in proteins and showed that it is not supported by the currently available scientific evidence [12]. Despite shortcomings in evidence, the notion of glyphosate for glycine substitution in proteins has attracted considerable interest and continues to be cited in the scientific literature and used in debates on glyphosate toxicity [13–15].

In order to resolve the controversy surrounding the assertion that glyphosate can substitute for glycine, we have conducted a proteomics analysis of mammalian cells grown in the presence of a high concentration of glyphosate. As proteomics employs a mass spectrometry approach, it can accurately measure a potential shift in the molecular weight of peptides derived from proteins, which could arise from the incorporation of amino acid variants [16], thus directly testing whether glyphosate for glycine substitution takes place.

## Main text

### Methods

#### Cell culture

Hormone-independent MDA-MB-231 human breast cancer cells were maintained in 75 cm^2^ flasks (Corning, Tewksbury, USA) as previously described [18]. Cells were seeded at 10^6^ cells in 75 cm^2^ flasks containing 10 mL Dulbecco’s Modified Eagle Medium (DMEM)-based maintenance medium. After a 24 hour (h) recovery period, cells were washed 3 times with 5 mL phosphate buffered saline (PBS), fresh medium added either with or without 100 mg/L glyphosate (Sigma-Aldrich Co Ltd, Gillingham, Dorset, UK), and culture continued for a further 6 days. Medium was refreshed at 24 and 96 h from the 1st day of treatment. The experiment was conducted in three biological replicates (3× negative controls and 3× glyphosate 100 mg/L, each condition in two technical replicates).

#### Sample preparation

Samples were lysed (PBS, 4% sodium dodecyl sulphate (SDS), 25 mM Tris(2-carboxyethyl)phosphine (TCEP), 1× complete ethylenediaminetetraacetic acid (EDTA)-free protease inhibitors (Roche Products Limited, Welwyn Garden City, UK) with 3 * 5 second (s) sonication on ice followed by heating at 95 °C for 10 minutes (min). Samples were alkylated with *N*-ethylmaleimide (50 mM) in the dark for 30 min at room temperature, centrifuged at 17,000*g* and the pellet discarded. Proteins were precipitated (with methanol–chloroform), dissolved in 100 µL of 0.1 M tetraethylammonium bromide (TEAB), 8 M urea, diluted 1:4 (urea to 2 M) and digested at 37 °C overnight with LysC (Wako Chemicals Europe, Neuss, Germany). Samples were further diluted 1:2.5 (urea 0.8 M) and digested at 37 °C for 16 h with trypsin (Thermo Fisher, Loughborough, UK). The digestion was stopped by adding trifluoroacetic acid (TFA) to a final concentration of 1%. Digested peptide samples were desalted using a tC18 SepPak plate (Waters UK, Elstree, UK), and 100 µg of each were labelled with Tandem Mass Tag (TMT)-6plex (Thermo Fisher). Labelled peptides were combined, dried, reconstituted in 1% TFA, desalted again as above, dried, and reconstituted in 5% formic acid.

#### Mass spectrometry analysis

The TMT labelled sample was analysed by RPLC-MS/MS/MS (145 min linear gradient) on a Fusion Tribrid Orbitrap operating in Data Dependent Acquisition mode (MultiNotch Simultaneous Precursor Selection method; MS1: profile mode, Orbitrap resolution 120 k, 400–1600 m/z, AGC target 400,000, 100 milliseconds (ms) maximum injection time, RF lens 60%; MS2: centroid mode, IonTrap, 10 dependent scans, 1.2 Th isolation window, charge states 2–6, 60 s dynamic exclusion, CID fragmentation (35%, activation Q 0.25), AGC target 10,000, 70 ms maximum injection time; MS3: profile mode, 5 precursors, 2 Th isolation window, Orbitrap resolution 30 k, 100–500 m/z, AGC target 50,000, 105 ms maximum injection time, HCD fragmentation (55%). The mass spectrometry proteomics data have been deposited to the ProteomeXchange Consortium via the PRIDE partner repository with the dataset identifier PXD013744.

#### Data analysis

The acquired raw file was searched with MaxQuant (1.6.0.13) against a human proteome Fasta database downloaded from UniProtKB. Because there was a single file to search, this allowed more variable modifications than normal without the search becoming impractically long. Variable modifications included in the search were “M-oxidation”, “N-terminal acetylation”, “deamidation (NQ)”, “Gln → pyroGlu”, “Phospho (STY)”, as well as two putative glyphosate-induced modifications: “A1” = glyoxylate-modified cysteine (+H_2_O_3_C_2_ => expected monoisotopic mass shift +74.0003939305 Da) and “A2” = Glycine replaced by glyphosate (+H_3_O_3_CP => expected monoisotopic mass shift +93.9819804726 Da); finally, “*N*-ethylmaleimide” was unusually set as a variable modification (no fixed modifications) since it would compete with modification “A1” for the same sites. All false discovery rates (FDRs) were set to 1%. Dependent peptide search was ticked. Following MaxQuant analysis, data was reprocessed starting from the level of individual evidences for modified peptides (peptidoforms) using DC Biosciences’ TMT-labelled data processing scripts. Briefly, the Levenberg–Marquardt procedure was applied by column to normalise samples. Peptidoform reporter intensities were calculated as the sum of those of individual evidences and re-normalised as above. Peptide ratios were calculated (glyphosate vs average control), re-normalised as above, and summarized at protein groups level using an in house, mean based algorithm. Protein groups with a single identified peptidoform were excluded from the analysis. Moderated Welch’s t-tests were calculated and, in order to address the multiple hypothesis testing problem, p-value significance thresholds for 10, 20 and 30% False Discovery Rate were identified using the Benjamini–Hochberg procedure. Thresholds for significant ratios (fold change) were defined as the 5% upper and 5% lower tails of the ratios between individual controls and average control samples.

### Results

In this study we set out to answer three distinct questions. First, are there any statistically robust global proteome changes in response to glyphosate treatment of mammalian cells? Second, can we identify modifications of cysteine residues as a result of the (putative) presence of glyoxylate, which is produced if glyphosate is metabolised (Fig. 1) [17]. Third, and most crucially, can we identify peptides where glyphosate has been directly incorporated in place of glycine?

**Fig. 2:**
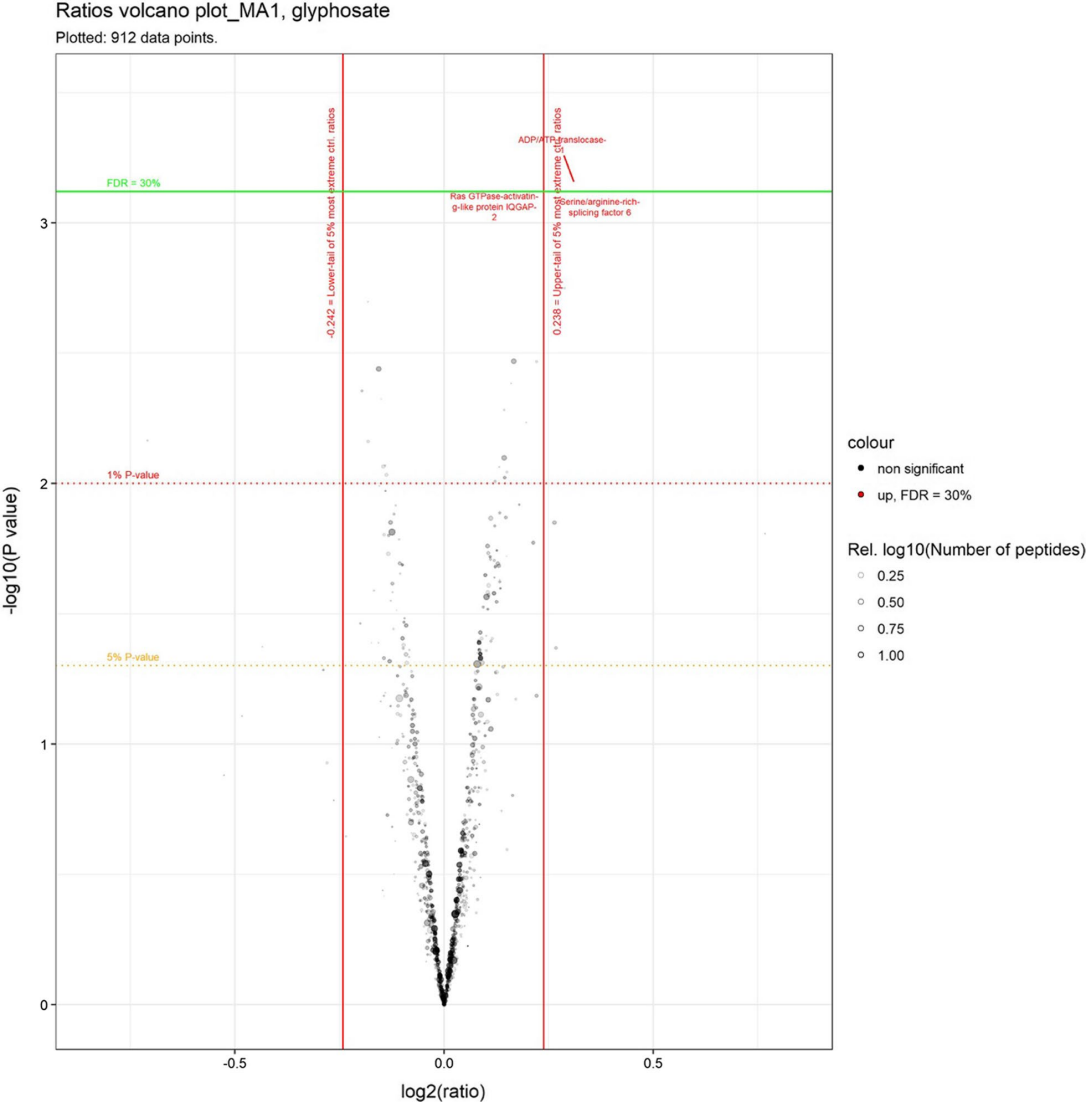
Volcano plot analysis of global proteomics changes after glyphosate treatment. X and Y axis: normalised log2 ratio and − log10 p-value of moderated Welch’s t-test. Vertical thresholds: upper and lower tails of the control-to-control ratios (5% most extreme log2 ratios in absolute value). Horizontal threshold: 30% False Discovery Rate (FDR) based on the Benjamini–Hochberg-procedure; there were no significant values at 10% and 20% FDR. Each dot represents a single protein group

Our results first confirmed our previous observation [18] that treatment of MDA-MB-231 cells with 100 mg/L glyphosate did not alter their growth characteristics (Additional file 1: Table S1). Statistical analysis of the ratios of global proteome changes between glyphosate treated and non-treated samples did not show significant changes (Fig. 2). Only two protein groups were found to be significantly up regulated; ADP/ATP translocase and serine/arginine-rich splicing factor 6. However, first these proteins are barely beyond the set thresholds and second, we would expect a small number of significant proteins under the null hypothesis with the criteria used.

**Fig. 3:**
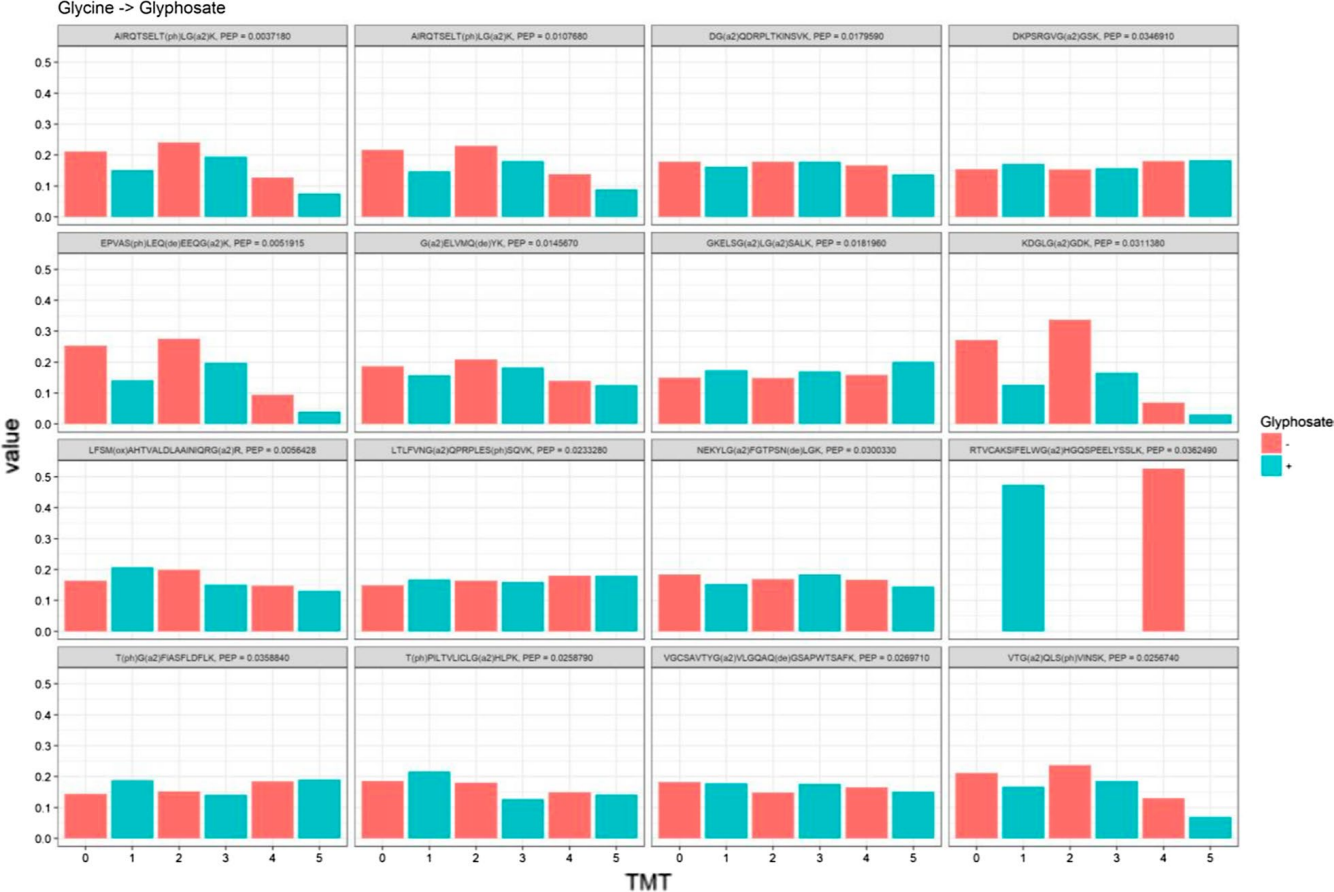
Normalised TMT reporter intensities per TMT channel for all putatively identified glycine for glyphosate substituted peptides (indicated by “G(a2)” in the modified sequence). Since some such peptide discoveries would be expected under the null hypothesis (no substitution), we designed the experiment to use the isobaric pattern as a validation. Samples order is ∓ Glyphosate for replicate 1, then 2, then 3. True discoveries would be expected to have null or only trace reporter intensities in red (untreated) channels, compared to strong signal in blue (treated) channels. The data conclusively shows that all candidate substituted peptides are false discoveries

We then tested samples for two different post-translational modifications (PTMs), which have been proposed to result from glyphosate exposure. Confident identification of new PTMs in proteomics is difficult, because peptide identification relies on matching rather than on full sequencing; peptide spectra are usually both hybrid and incomplete, so that spectra which can be fully de novo sequenced are rare. Proteomics peptide searches typically work at 1% FDR, which means that for any PTM, however unlikely to be truly present in the samples, it is to be expected that some peptides will nonetheless be identified. Normally, careful verification of putative PTMs is thus required, including analysis of synthetic peptides to show that their spectrum is similar to that of the identical, putative identifications. In this experiment, however, neither of the two putative PTMs of interest would be expected to be present in absence of glyphosate treatment. It was thus possible to use TMT labelling to identify and filter out any potential false discoveries. Indeed, by combining three treated and three untreated samples as one TMT-6plex labelled sample, we would expect a specific pattern (+/+/+/−/−/−) of reporter intensities for peptides bearing true glyphosate treatment induced-PTMs. By contrast, we would expect this pattern to only occur very rarely for peptides not bearing these PTMs: these would be putative peptides from proteins whose abundance would increase massively as a result of glyphosate treatment. However, as discussed above global analysis of the samples’ proteome failed to showcase any significant proteome changes as a response to treatment. Thus, in this experiment the pattern of peptides’ TMT reporter intensities constitutes a string filter to segregate real hits from false discoveries. Only glycine to glyphosate candidate peptides were identified in the search. As shown in Fig. 3, analysis of the TMT reporter intensity pattern of all candidates shows conclusively that they are all false discoveries, as none display the expected TMT pattern. True discoveries would be expected to have null or only trace reporter intensities in untreated channels (Fig. 3, red histogram bars), compared to a strong signal in treated channels (Fig. 3, blue histogram bars). Thus, we can confidently conclude that this analysis did not provide any evidence for the occurrence of either glyoxylation or substitution of glycine for glyphosate in proteins.

### Discussion

We provide here a direct test using a proteomics approach of the assertion that glyphosate substitutes for glycine in proteins of mammals [10, 15]. Our results clearly show that glyphosate does not substitute for glycine in peptide chains (Fig. 3), which is in accord with previous observations in bacteria [16, 20]. In addition, our experiment allowed testing of glyphosate’s effects on the proteome profile of MDA-MB-231 cells and if the putative glyphosate metabolite glyoxylate could cause modifications of cysteine residues. No statistically significant effects were detected (Figs. 2, 3), which suggests that glyphosate does not have an effect on the proteome at the concentration tested.

In conclusion, our proteomics analysis proves the claim that glyphosate can substitute for glycine in proteins negatively affecting their structure and function is incorrect. Although our results will not come as a surprise to most of the scientific community, we believe they are nonetheless important in helping to clarify the debate on glyphosate toxicity in which many scientific hypotheses are considered as evidence of harm, ultimately influencing political debates, without being carefully tested in a controlled laboratory setting. We thus hope that our study will assist in focusing researchers’ attention on other aspects of glyphosate safety profiles, which remain to be investigated such as its impact on reproduction, development, carcinogenicity and microbiomes, especially of the gut [21–23].

## Limitations

Our inability to find glyoxylation of proteins is not unexpected since there is little or no evidence to show that glyphosate can be metabolised to glyoxylate and aminomethylphosphonic acid in mammals. Only a single study has shown glyphosate-derived glyoxylation of proteins where mice were administered with a very high dose of glyphosate (200 mg/kg body weight) with unknown health implications [17].

As our results were generated using a single cell line, this can question the generalisation of the findings presented and the efficiency of glyphosate uptake. However, as our investigation is focused on the function of the mRNA translation machinery, which is the same in all mammalian cell types our findings in MDA-MB-231 cells are very likely generally applicable. The choice of MDA-MB-231 cells and glyphosate concentration is based on our previous studies, which showed that 100 mg/L glyphosate did not result in cytotoxicity or growth inhibition [18], which we also observed here (Additional file 1: Table S1). The concentration of glyphosate tested (100 mg/L; 0.59 mM) was chosen so that it was slightly higher than the concentration of glycine (30 mg/L; 0.4 mM) in the DMEM-based culture medium. Furthermore, a previous study where HepG2 cells were treated with 45 mg/L ^14^C-glyphosate for a 24 h period, found that 20% of this compound entered these cells [19]. Thus, it can be expected that glyphosate at the higher concentration used here will be readily taken up by MDA-MB-231 cells.

## Additional file

**Additional file 1: Table S1.** Kinetics of MDA-MB-231 cell growth in either the presence or absence of 100 mg/L glyphosate. Cell counts are given at day-1 of seeding flasks and following 6-days of continuous culture. Note: no differences in cell numbers were observed between negative control and glyphosate treated cultures.

## Data Availability

The mass spectrometry proteomics data have been deposited to the ProteomeXchange Consortium via the PRIDE partner repository with the dataset identifier PXD013744.

## Abbreviations

PBS: phosphate buffered saline
SDS: sodium dodecyl sulphate
TCEP: Tris(2-carboxyethyl)phosphine
EDTA: ethylenediaminetetraacetic acid
TEAB: tetraethylammonium bromide
TFA: trifluoroacetic acid
TMT: tandem mass tag
PTM: post-translational modification
FDR: false discovery rate
DMEM: Dulbecco’s Modified Eagle Medium
